# Gastric cancer associated variant of DNA polymerase beta (Leu22Pro) promotes DNA replication associated double strand breaks

**DOI:** 10.18632/oncotarget.4426

**Published:** 2015-06-10

**Authors:** Jenna Rozacky, Antoni A. Nemec, Joann B. Sweasy, Dawit Kidane

**Affiliations:** ^1^ Division of Pharmacology and Toxicology, College of Pharmacy, The University of Texas at Austin, Dell Pediatric Research Institute, Austin, TX, USA; ^2^ Department of Biomedical Sciences, Florida State University College of Medicine, Tallahassee, FL, USA; ^3^ Departments of Therapeutic Radiology and Genetics, The Yale Comprehensive Cancer Center, New Haven CT, USA

**Keywords:** DNA polymerase beta, gastric cancer

## Abstract

DNA polymerase beta (Pol β) is a key enzymefor the protection against oxidative DNA lesions via itsrole in base excision repair (BER). Approximately 1/3 of tumors studied to date express Pol β variant proteins, and several tumors overexpress Pol β. Pol β possesses DNA polymerase and dRP lyase activities, both of which are known to be important for efficient BER. The dRP lyase activity resides within the 8kDa amino terminal domain of Pol β, is responsible for removal of the 5′ phosphate group (5′-dRP). The DNA polymerase subsequently fills the gaps. Previously, we demonstrated that the human gastric cancer-associated variant of Pol β (Leu22Pro (L22P)) lacks dRP lyase function *in vitro*. Here, we report that L22P-expressing cells harbor significantly increased replication associated DNA double strand breaks (DSBs) and defective maintenance of the nascent DNA strand (NDS) during replication stress. Moreover, L22P-expressing cells are sensitive to PARP1 inhibitors, which suggests trapped PARP1 binds to the 5′-dRP group and blocks replications forks, resulting in fork collapse and DSBs. Our data suggest that the normal function of the dRP lyase is critical to maintain replication fork integrity and prevent replication fork collapse to DSBs and cellular transformation.

## INTRODUCTION

Base excision repair (BER) is considered a major DNA repair system in mammalian cells that corrects up to 20,000 lesions per cell per day caused by reactive oxygen species (ROS) and reactive nitrogen species (RNS) [1-3]. In addition, BER is an important DNA repair pathway protecting mammalian cells against single-base DNA damage caused by methylating and oxidizing agents and spontaneously arising apurinic/apyrimidinic (AP) sites [4, 5]. It has been proposed that BER proceeds via a highly orchestrated mechanism in which the enzyme involved at one step directly communicates with the enzyme of the next [6]. DNA glycosylases remove a damaged base through hydrolysis of the glycosidic bond linking the damaged base to the sugar generating an AP site [7]. Normally, repair of an AP site proceeds through hydrolysis by AP endonuclease of the phosphodiester bond 5′ to the abasic site [8, 9]. This is followed by a coordinated reaction in which DNA polymerase β (Pol β) adds one nucleotide to the 3′ end of the incised AP site, simultaneously removing a 5′-sugar phosphate (5′-deoxyribosephosphate; 5′-dRP) residue by β-elimination [10, 11]. The repair cycle is completed by the concerted action of AP-endonucleases, Pol β and DNA ligases [5].

Pol β is a major repair DNA polymerase that participates in short and long-patch repair of AP sites generated by glycosylases [12]. Pol β is bi-functional in BER, possessing both an 8kDa N-terminal domain containing 5-deoxyribose phosphate lyase and single-strand DNA binding activities, and a 31 kDa C-terminal domain that contains catalytic activity [10], which lacks 3′–5′ exonuclease activity, with the proofreading function attributed to autonomous exonucleases such as APE1 [13]. Previously, APE1 was shown to stimulate the dRP activity of Pol β [14], and this could influence the formation of repair products since the removal of the dRP group is considered to be a rate-limiting step in short patch BER and is required for ligation of the BER intermediates after gap-filling by a polymerase [15]. Elimination of the dRP lyase activity of Pol β, or the entire *POLB* gene, leads to increased sensitivity to DNA-damaging agents [16], genetic instability [17], and neonatal lethality [18] respectively.

Previous studies have shown a correlation between single nucleotide polymorphisms (SNPs) of the *POLB* gene and the risk to develop various cancers, including gastric cancer [19, 20]. There is evidence that some of the *POLB* polymorphisms found in cancer cells correlate with defects in the repair of DNA damage induced by several anti-cancer agents[21]. For example, results from small-scale studies have shown that about one third of all human tumors express Pol β variant proteins [22] and some of these tumor-associated variants induce a mutator phenotype [19, 23], genomic instability and cellular transformation [24]. Pol β containing the L22P mutation in the dRP lyase domain has been identified in cells derived from a gastric carcinoma [25, 26]. The mutation was found to significantly impair both enzymatic activities; Pol β (L22P) exhibits negligible 5′-deoxribose phosphate (dRP) lyase activity, and very low [26] or no [27] polymerase activity. Molecular dynamics simulations indicated that the L22P mutant is characterized by altered packing that results in considerable destabilization [26]. Although L22P is not directly involved in forming the DNA binding pocket, it has decreased DNA binding affinity.

The mutation may alter the organization of the binding pocket, preventing Pol β from binding DNA efficiently and preventing polymerization from occurring. Hence, any mutations in the dRP lyase domain, whether or not they are in critical residues, can prevent the enzyme from participating in BER. In vivo L22P mutation could prevent the removal of the 5′-dRP group and the filling of the gap. They could also prevent Polβ from binding the DNA that would result in unrepaired lesions. These variants could result in an accumulation of BER intermediates leading to genomic instability. Given the large size of the mammalian genomes, DNA replication is a process that is tightly monitored [28]; however, L22P mutation may threaten genome integrity by interfering with progression, stability, and proper resumption of replication after fork arrest. Unrepaired DNA can result in stalled and collapsed replication forks leading to the formation of DSBs. However, the impact of dRP lyase deficiency on replication fork progression or stability is not yet established. Defects of DNA replication or failure to restart stalled forks can lead to accumulation of mutations and genomic aberrations [28]. In this study, we investigated the mechanism of how the dRP lyase-deficient gastric cancer variant of Pol β (L22P) induces replication associated DSBs to promote genomic instability and cellular transformation. In addition, our study confirmed that treatment with a PARP1 inhibitor eliminates L22P expressing cells via trapping a PARP1 5′-dRP group complex which suggests that trapped PARP1 may likely blocked replication forks that ultimately leads to DSBs.

## RESULTS

### Double strand breaks increase in cells expressing the L22P variant of gastric cancer

**Figure 1:**
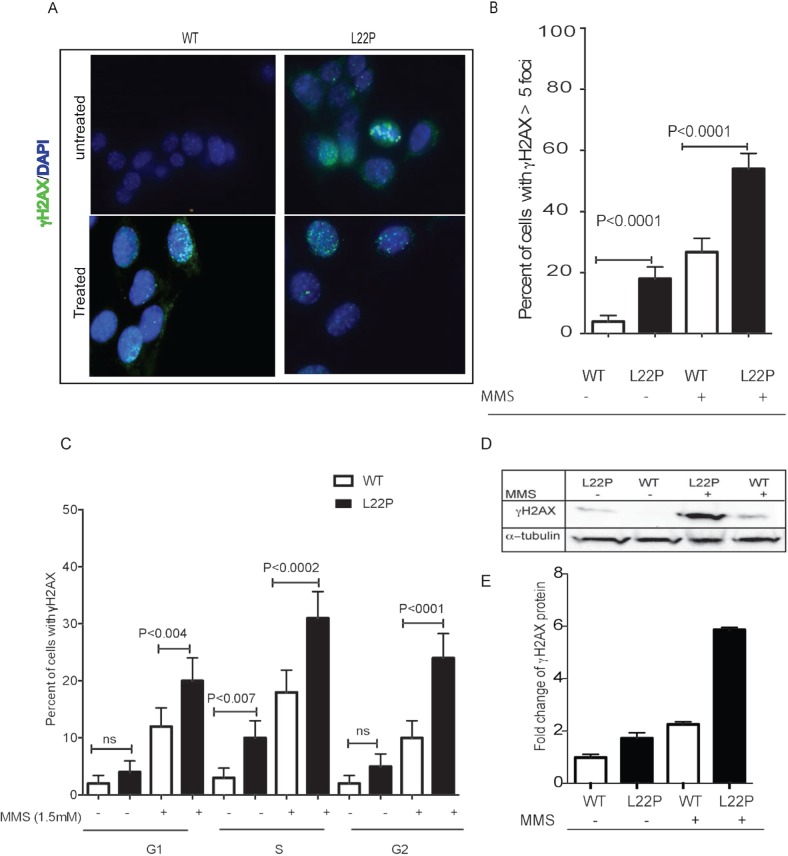
L22P-expressing cells induced S-phase dependent DSBs **A.** Representative images of DNA double strand breaks in cells expressing L22P and wild type of DNA polymerase beta before and after MMS treatment. **B.** Quantified percentage of γH2AX ( > 5 foci) and analyzed using GraphPad Prism. **C.** Estimated percentage of cells with γH2AX in different cell cycle stages based on FACS analysis before and after one-hour treatment of 1.5mM MMS. Cells were stained with γH2AX antibody and propidium iodide to assess the levels of double-strand breaks (DSBs) and the cell cycle phase, respectively, and analyzed by flow cytometry. **D.** Western blot analysis of γH2AX before and after 1 hour of 1.5mM MMS treatment. **E.** Estimated fold change of γH2AX proteins in MMS treated versus untreated cells from three different experiments. The level of γH2AX increased ~2 and ~3 fold in untreated versus treated L22P-expressing cells than WT cells. Note that quantification of bands was carried out using the NIH Image J program by measuring the integrative density of each γH2AX band that is normalized with alpha-tubulin band as internal control. All data analyzed using GraphPad prism.

To determine the susceptibility of L22P-expressing cells to DSBs, we stained L22P-expressing and wild-type Pol β (WT) cells for histone H2AX that is rapidly phosphorylated in the chromatin microenvironment surrounding DSBs [29]. Surprisingly, we noticed that the levels of spontaneous DSBs increased significantly in the L22P-expressing cells versus WT cells (Figure 1A Mean ± SEM; 14±3.5; *P* < 0.001). We expanded our study to determine whether treatment with the alkylating agent methylmethane sulfonate (MMS) exacerbated the formation of DSBs in cells expressing the L22P variant of Pol β. We counted the number of cells with γH2AX foci greater than five foci per cells after the cells were treated with 1.5 mM MMS for one hour in L22P (*n* = 92) and WT cells (*n* = 96) (Figure 1A). We found that the number of cells with γH2AX foci were increased significantly in L22P-expressing cells versus WT expressing cells treated with MMS (Figure 1A and 1B; Mean ±SEM; 25±4; *P* < 0.001). Next, we determined the distribution of DSBs in different stages of cell cycle, and we found that spontaneous DSBs are increased significantly during S-phase in L22P compared to WT cells (*P* < 0.001; Figure 1C). In contrast, the number of DSBs in untreated WT versus L22P-expressing cells was not statistically significant during the G1 and G2 stages of cell cycle. In addition, DSBs are increased significantly in L22P treated versus WT cells in all stages of the cell cycle (G1, *P* < 0.004; S, *P* < 0.0002; G2, *P* < 0.0001, Figure 1C). Furthermore, we found that the level of γH2AX proteins increased ~2- and ~3-fold in untreated versus treated L22P-expressing compared with WT, respectively (Figure 1D and 1E).

### L22P induces replication associated DNA double strand breaks

**Figure 2:**
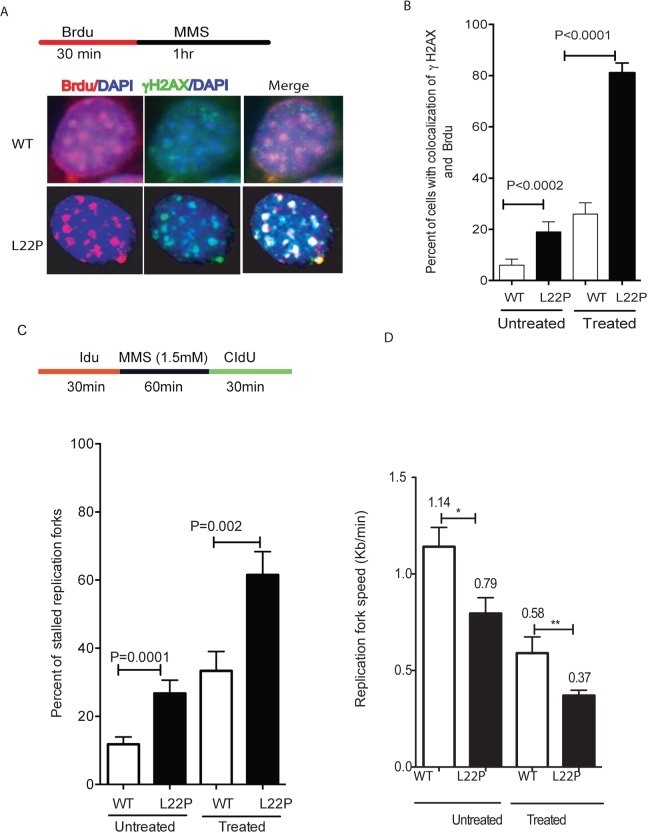
Replication fibers and fork rates in wild type (WT) and L22P-expressing GES-1 cells **A.** Representative images of co-localization of γH2Ax and Brdu positive cells. **B.** Estimated number of cells with co-localization of γH2Ax and Brdu positive WT (*n* = 143) and L22P-expressing cells (*n* = 160) treated with MMS versus untreated WT (*n* = 97) and L22P (*n* = 102). Double positive cells (γH2Ax +Brdu) in L22P-expressing cells are a statistically significant difference compared to WT (*P* < 0.001). **C.** Estimated percentage of replication fork stall in L22p expressing cells and WT GES-1 cells. Note that the schematic representation of replication tracts in WT and L22P-expressing GES cells were first labeled with Idu (25μm) for 30 minutes (red line), then treated with 1.5 mM MMS for 1 hour (black line) followed by a second labeling with CIdU (250μm) for 30 minutes (green) then processed for DNA fiber spread as described in Materials and Methods. **D.** Estimated replication fork speed in cells expressing L22P and WT before and after treatment with 1.5mM MMS and HU.

Although the findings above establish that DSBs are formed at a significantly higher level in L22P-expressing cells compared to WT cells, the mechanism of how DSBs are generated is not known. In order to determine whether the DSBs are associated with actively replicating DNA, we performed co-immunostaining of the cells with antisera against Brdu and γH2AX. The majority of MMS-induced γH2AX foci were present in Brdu-positive cells, and thus these foci are attributed to replication-mediated DNA damage in L22P-expressing cells versus WT cells during S-phase (Figure 2A). The number of double-positive cells (Brdu+ γH2AX) was significantly higher in L22P-expressing cells than in WT cells (Figure 2B; *P* < 0001). Furthermore, we have evaluated L22P expression-induced replication-dependent DSBs using the DNA combing assay [30]. The general schematic for these experiments is shown in Figure 2C. The replication fork speed after treatment was quantified by dividing the length of each fluorescent track by the time of incubation with the halogenated nucleotide, as shown in Figure 2C. Interestingly, we found that L22P-expressing cells spontaneously exhibited 26.7% stalled forks which increases up to 61.5 % after one hour of MMS treatment (Figure 2C) as compared to treated WT cells (33%). In addition, the speed of the replication forks was reduced to 0.37kb/min in treated L22P-expressing cells compared to 0.58kb/min in WT cells (Figure 2D). Surprisingly, we observed significant delay in spontaneous fork progression in L22P-expressing cells versus WT cells (0.79kb/min; 1.14kb/min; *P* < 0.01; Figure 2D).

### L22P is deficient in maintenance of nascent DNA strand (NDSs) during replication stress

**Figure 3:**
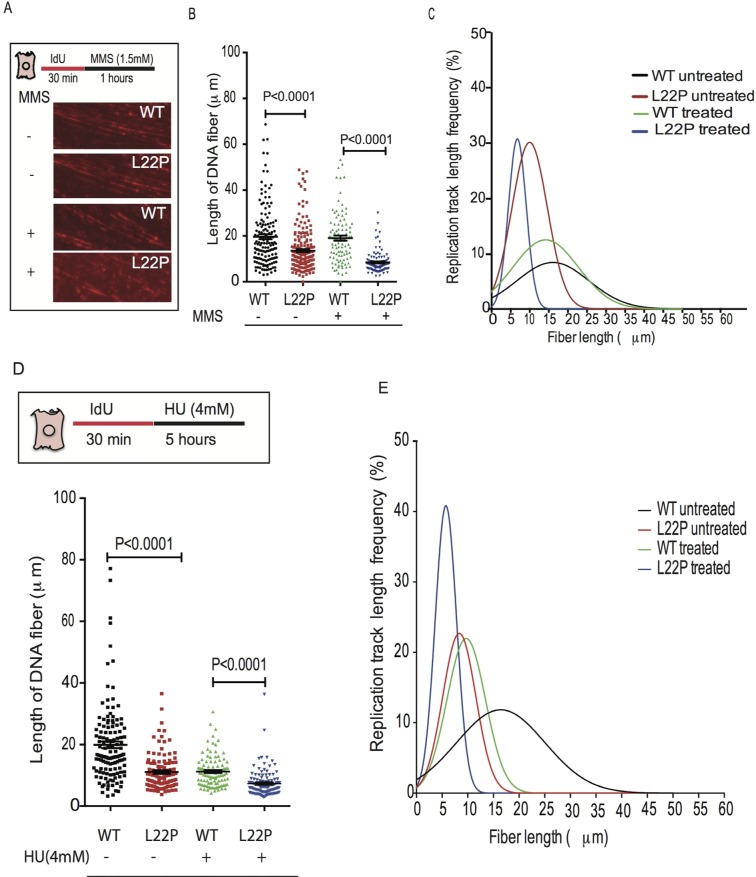
Normal function of DNA polymerase beta is required for maintenance of nascent DNA after DNA replication stress The WT and L22P-expressing cells were labeled with IdU for 30 minutes and then followed with 1 hour of MMS or 5 hours of HU treatment. **A.** Representative images of wild type and L22P-expressing cells pulsed with IdU for 30minutes and treated for 1hr with MMS or untreated **B.** Estimated length of fiber in wild type and L22P-expressing cells with and without MMS treatment **C.** The replication track length frequency (%) analyzed based on Gaussian distribution (nonlinear regression, curve fit) for treated WT and L22P expressing cells (*n* = 101; 100) and WT and L22P expressing cells (*n* = 146; 201) without MMS treatment. **D.** Quantified length of fiber for wild type and L22P-expressing cells pulsed with IdU for 30 minutes treated with or without HU (4mM) for 5 hours. **E.** Distribution of replication track length frequency in HU treated WT (*n* = 109) and L22P cells (*n* = 119) versus the number of cells in untreated WT (*n* = 132) and L22P-expressing cells (*n* = 107). The cumulative percentage of replication track length frequency is calculated by dividing the total frequency of replication track length of NDS ≤10μm by the total frequency of replication track length of NDS ≤50μm then multiplied by 100. Note that the solid black rectangle represents WT untreated cells; solid red rectangle represents untreated L22P expressing cells; green triangle represents treated wild type and blue color inverted triangle represents treated L22P-expressing cells. All data analyzed using GraphPad prism.

To determine whether the dRP lyase activity is critical for maintenance of NDS during replication stress, we used a single-molecule DNA fiber technique [31]. For each experimental condition, individual DNA fibers were uniformly stretched on glass coverslips, and visualized by fluorescence microscopy using specific antibodies to detect IdU labeled DNA (Figure 3A). First, we determined that MMS-mediated DSBs induced instability of the NDS in dRP lyase-deficient compared to WT cells. We found that the length of the NDS was significantly shorter in L22P-expressing versus WT cells (Figure 3B, Mean ± SEM, 8±0.4; 19±1.1; *P* < 0.0001). On the other hand, ~84% of the cumulative percentage of replication track length frequency ranged between 1-10μm in L22P as compared to WT cells (53%) (Figure 3C) (Cumulative percentage of replication track length frequency is calculated by dividing the total frequency of replication track length ≤10μm by the total frequency of replication track length ≤50μm then multiplied by 100). Interestingly, we noticed that DNA fibers that contained only IdU tracts were significantly shorter in HU-treated L22P-expressing cells versus HU-treated WT cells (Figure 3D Mean ± SEM; 7.3±0.4μm and 11±0.5 μm, *P* < 0.0001), implying that the collapsed replication forks were not maintained in L22P-expressing cells. In addition, to determine how L22P expression affects the normal distribution of nascent DNA stability, we analyzed the data based on the cumulative percentage of the replication track length frequency. We found that 61% and 22% of replication track length was observed in range of 1-10μm in untreated L22P-expressing cells versus wild type cells, respectively. However, 5 hours of treatment with HU increased the cumulative percentage of replication track length frequency to 87% in L22P-expressing cells versus WT cells (Figure 3E). These results suggest that the dRP lyase domain of DNA polymerase beta is critical for the maintenance of NDSs in response to replication-associated DSBs.

### dRP lyase-deficient cells increase RPA coated ssDNA and the appearance of Rad51

**Figure 4:**
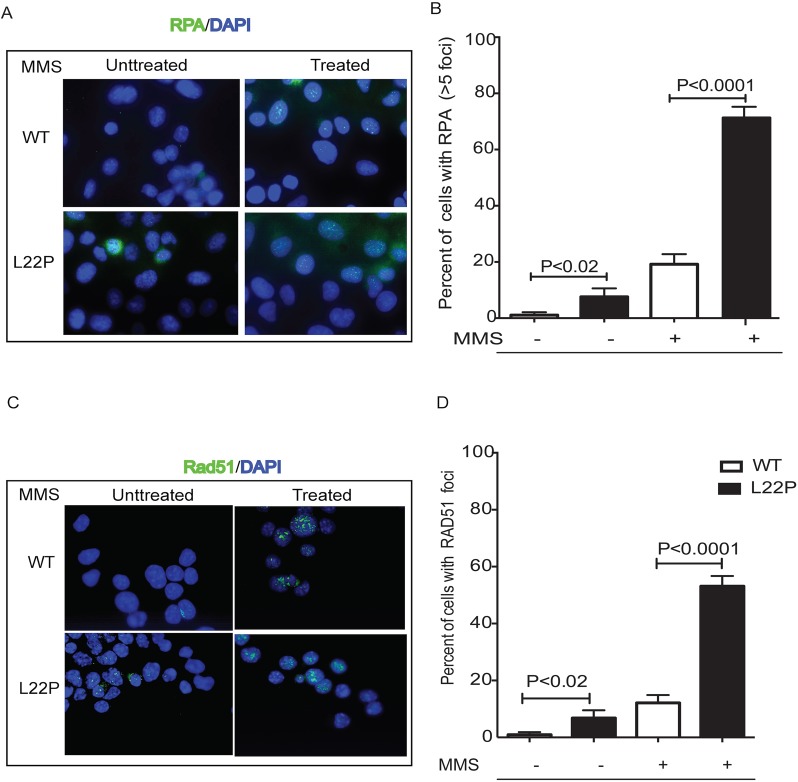
Immunofluorescence analysis of Rad51 foci in L22P-expressing cells before and after MMS treatment Wild type and L22P-expressing cells were treated with 1.5mM mms for 1 hour and then allowed to repair for 6 hours. **A.** RPA localization in wild type and L22P-expressing GES-1 cells before and after 1.5mM MMS treatment. **B.** Quantification of number of cells with RPA foci greater than 5 foci per nuclei. Note that the number of untreated wild type (*n* = 96) and L22P expressing cells (*n* = 79) versus treated WT (*n* = 120) and L22P-expressing cells (*n* = 136) included for analysis. **C.** The spatial distribution of Rad51 foci is shown in representative nuclei of wild type and L22P-expressing cells before and after treatment. **D.** Quantification of the percentage of Rad51 foci. Cells with at least 10 foci were counted as Rad51 positive cells and analyzed with GraphPad Prism software. Note that the number of untreated WT (*n* = 108) and L22P expressing cells (*n* = 88) versus treated WT cells (*n* = 140) and L22P expressing cells (*n* = 192) included in this analysis.

The formation of γH2AX foci in dRP lyase-deficient Pol β cells prompted us to analyze the Rad51 and RPA protein localization. Replication protein A (RPA), the major single-stranded DNA (ssDNA)-binding protein in eukaryotic cells, accumulates along stretches of ssDNA generated by stalled replication forks and/or DNA damage [32]. To determine if ssDNA levels increased during MMS treatment in L22P-expressing cells, we conducted immunofluorescence experiments and localized RPA foci (Figure 4A). Our data shows that the number of cells with RPA foci in treated cells were significantly increased (*n* = 143, 63%), versus non-treated controls (*n* = 176, 18%) (*P* = 0.001) (Figure 4B). Once DSBs are induced by MMS treatment, 5′-DNA end resection of DSBs is a prerequisite for loading of Rad51 to promote strand exchange activity [33]. When the cells were analyzed for the presence of RAD51 foci (Figure 4C), 50% of the infected cells exhibited foci compared to 13% for non-infected controls (13%; *n* = 67) (*P* = 0.0001) (Figure 4D).

### L22P induces chromosomal aberrations and cellular transformation

**Figure 5:**
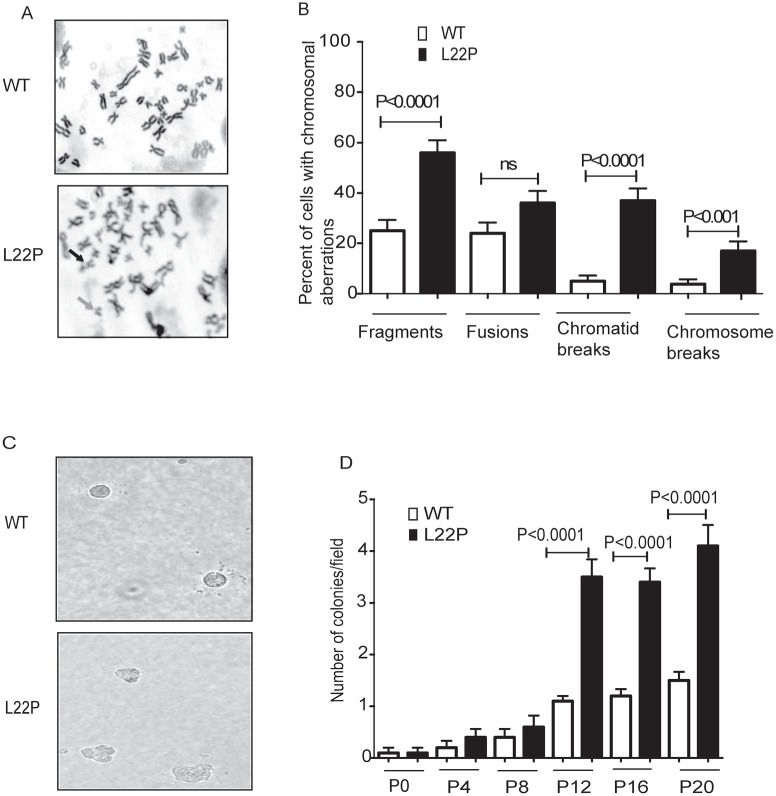
L22P induces chromosomal aberrations and cellular transformation **A.** Metaphase spread from wild type (*n* = 75) and L22P (*n* = 75) cells. Chromosomal fragmentation, fusions, Chromatid breaks (SSB) and chromosomal breaks (DSB) highly dominant in cells expressing L22P than wild type. Note that black arrows show chromosome breaks and gray arrows show fragments. **B.** Quantified percentage of chromosomal aberration are significantly different in L22P-expressing cells than WT (*P* < 0.0001). **C.** Representative image of transformed cells after anchorage-independent growth of GES-1 cells expressing L22P and WT-Pol β at 20x magnification. **D.** Number of colonies per field are plotted on Y-axis. Cells were scored at passage (P) 0, 4, 8, 12, 16, and 20 after 5 weeks of growth in soft agar.

We have shown that following treatment with MMS, DSBs arise in a replication-dependent manner when replication forks encounter BER intermediates that are generated via L22P. Here, we determined whether cells deficient in dRP lyase function are vulnerable to chromosomal aberrations because of a deficiency in removing the 5′dRP, as this group may yield single-nucleotide gaps on duplex DNA due to incomplete BER. To test the hypothesis that a deficiency in dRP lyase of Polβ leads to an increase in chromosomal aberration frequency, we prepared metaphase spreads from normal gastric epithelial cells that expressed the L22P variant of DNA Pol β (Figure 5A). Chromosomal aberrations such as chromatid breaks, chromosome breaks, fusions and fragments were evaluated and presented at significantly higher levels in the L22P-expressing GES-1 cells versus WT cells (*P* < 0.0001) (Figure 5B). In this study we determined that cells deficient in dRP lyase function are vulnerable to chromosomal aberration induction due to a deficiency in removing the 5′dRP group. Furthermore, we asked if expression of L22P induces cellular transformation. We expressed HA-tagged WT and L22P to equal levels in normal human gastric epithelial GES-1 pools cells and performed a soft agar assay in which cells that are able to grow in anchorage-independent manner on soft agar, while non-transformed cells are unable to grow and will not form colonies (Figure 5C). We found that cells expressing L22P formed significantly more colonies starting from passage 12 compared to WT demonstrating that expression of L22P induced a transformed phenotype (*P* < 0.001; Figure 5D).

### L22P expressing cells are sensitive to PARP1 inhibitors

**Figure 6:**
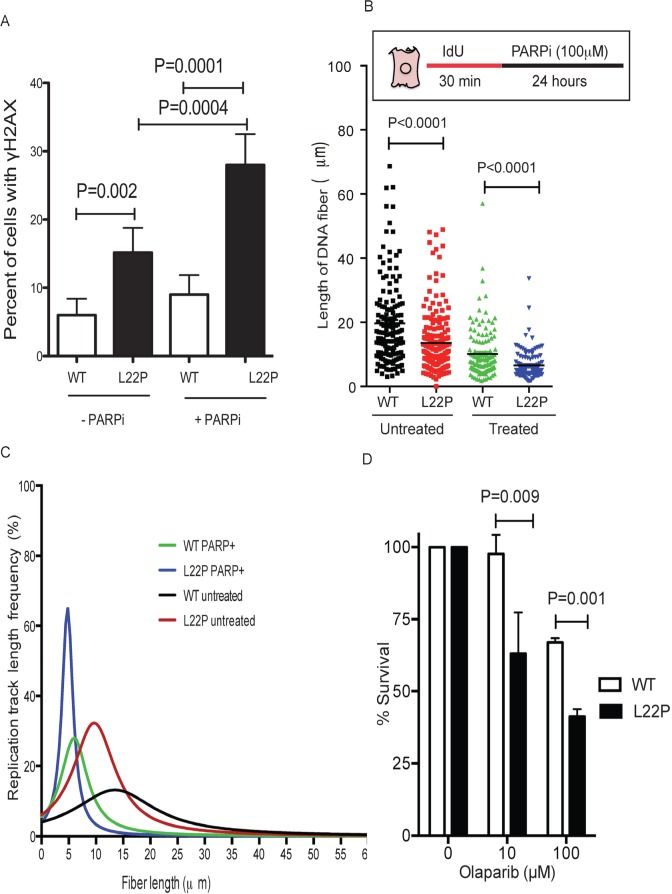
L22P variant of DNA polymerase beta confers sensitivity to PARP1 inhibitors compared to WT cells **A.** Estimated percentages of γH2AX in L22P and WT of cells treated with or without PARP1 inhibitor. **B.** The distribution of NDS in untreated WT (*n* = 110) and L22P-expressing cells (*n* = 120) and PARP1 inhibitor treated wild type (*n* = 123) and L22P-expressing cells (*n* = 122). Note that the schematic representation of replication tracts in WT and L22P-expressing GES cells were first labeled with Idu (25m) for 30 minutes (red line), then treated with 100mM PARP1 inhibitor ((PARPi) olaparib) for 24 hour (black line) then processed for DNA fiber spread as described in Materials and Methods. DNA fiber lengths were measured using NIH imageJ and data were analyzed using GraphPad Prism. **C.** The replication fork track length frequency in WT and L22P-expressing cells treated with PARP1 inhibitor. **D.** Clonogenic survival assays were conducted with GES-1 cells expressing WT or L22P Pol β in two different concentration of PARP1 inhibitor (10μM and 100μM). Data were analyzed two ways of ANOVA using GraphPad Prism.

Previous study suggests that PARP1 is associated with replication forks [34] and inhibition of PARP1 leads to stalled replication fork and the formation of DNA double strand breaks [35]. Here, we wanted to test whether PARP1 inhibitors synergize to increase DSBs in L22P-expressing cells. Our data shows that a significant increase in DSBs in L22P-expressing cells compared to WT cells (Figure 6A). Furthermore, we evaluated the stability of the NDSs in response to PARP1 inhibition in L22P-expressing cells. For this experiment, L22P-expressing cells and WT cells were pulsed with Idu for 30 minutes and then treated with a PARP1 inhibitor for 24 hours. Our data show that the average length of NDS is significantly decreased in L22P versus wild type cells treated with a PARP1 inhibitor (Mean ± SEM; 6.5 ± 0.4 versus 10± 0.7; *P* < 0.0001, Figure 6B). In addition, the average length of NDS significantly decrease in L22P-expressing cells versus wild type cells untreated with PARP1 inhibitor (13.5 ± 0.64 versus 19.6± 1; *P* < 0.0001). The cumulative percentage of frequency of track length greater than 10μm significantly decreased in L22P-expressing cells versus wild type cells (28% versus 53%; *P* < 0.0001, Figure 6C). To determine if expression of the L22P variant in the presence of WT sensitizes cells to PARP1 inhibitors, we conducted clonogenic survival assays using WT and L22P expressing GES-1 cells treated with low (10μM) and high (100μM) concentrations of PARP1 inhibitor. We found that treatment with a PARP1 inhibitor significantly decreased the percentage of cell survival in L22P-expressing cells (*P* < 0.01) (Figure 6D). In contrast, WT GES-1 cells in which the repair intermediate does not have the 5′-dRP group, and both PARP1 binding and inhibitor-mediated sensitization were minimal (Figure 6D). In combination with our chromosomal aberration studies, these results suggest that some of the cells harboring genomic instability are likely to survive and may undergo transformation (5B). Therefore, PARP1 inhibitors are likely a potential choice to treat L22P-expressing cells to suppress genomic instability and cellular transformation to eliminate precancerous cells.

## DISCUSSION

Previous reports indicated that the L22P variant of Pol β found in gastric cancer lacks dRP lyase activity and is sensitive to DNA damaging agents [26]. However, the mechanism by the lack of 5′-dRP causes genomic instability and cellular transformation is not known. In this study, we report evidence for the existences of replication-dependent DSBs that may cause genomic instability and cellular transformation in dRP lyase-deficient cells. We found that the number of γH2AX foci is significantly increased in L22P-expressing cells compared with the WT cells, suggesting that unrepaired 5′-dRP groups in L22P-expressing cells most likely leads to DSB formation. Even though spontaneous DSBs are significantly increased in L22P-expressing cells at S-phase (Figure 1C), monofunctional DNA methylating agents such as MMS increased the number of DSBs in all stages of the cell cycle similar to MMS treated Pol β-deficient cells [36]. Our data suggest that the dRP lyase activity is required to repair methylated base adducts in all stages of cell cycle.

We observed that MMS treatment increases the number of stalled forks and DSBs in S-phase in L22P-expressing cells versus WT (Figure 2C), suggesting that moving replication forks may encounter 5′-dRP group or other DNA lesions. Our data are in agreement with a previous report that found that DSBs after MMS arise when replication forks encounter BER intermediates such as N3-MeA [37]. Moreover, MMS-induced lesions reduced replication fork speed in L22P-expressing cells (Figure 2D) suggested that lack of dRP lyase function in L22P-expressing cells likely increases 5′-dRP groups and blocks DNA polymerase progression similar to previous study [38]. Alternatively, the replisome may encounter the 5′-dRP group and block the polymerases at replication fork. Our results indicate that replication-dependent DSBs arising after methylation damage involve replication fork stalling and collapse at methylated base adducts, likely similar to the mechanism of DSB formation after prolonged HU treatment [31, 39]. Our data are in agreement with previous studies that have indicated that BER intermediates are DNA synthesis-blocking lesions and are cytotoxic [16, 40]. Moreover, we found that after treatment with MMS or HU the length of NDSs were significantly reduced in L22P-expressing cells versus WT cells (Figure 3B and 3D). The shorter DNA could arise when the replication fork progression diminished after treatment or DNA strand at replication fork may prone to subsequent DNA nuclease attack in L22P-expressing cells than WT. This could suggest that dRP lyase is required for maintenance of NDSs during replication stress. On the other hand, many studies have shown that alkylating agents including MMS activate the DNA damage response pathway [41]. Collapsed replication forks in L22P cells likely utilize homologous recombination to restore active replication forks. Our data show that the number of cells with RPA and Rad51 are significantly increased in L22P-expressing cells which suggests that stimulation of homologous recombination may be involved in processing replication collapsed intermediates to stabilize the stalled replication fork or repair the collapsed fork.

Base lesions, abasic sites, and strand breaks all exhibit varying degrees of cellular toxicity, suggesting that targeting the 5′-dRP group may offer an additional avenue to increase sensitivity in gastric cancer cells. Previous reports show that modulating DNA glycosylase expression [42], blocking abasic site repair [43], or inhibition of PARP1 [44] offer alternative avenues for increasing sensitivity of cancer cells. We observed that inhibition of PARP1 increases sensitivity in L22P-expressing cells. Our results demonstrated that the L22P variant of Pol β may accumulate 5′-dRP groups key for PARP1 binding, such that in the absence of Pol β dRP lyase activity there is more likely PARP1 binding and more PARP1 inhibitor-induced cell killing (Figure 6). On the other hand, our data show that sensitivity in L22P-expressing cells for PARP1 inhibition may likely suggest that inhibited PARP1 results in cytotoxicity due to formation of replication-dependent DSBs [45]. In addition, PARP1 inhibitors induce loss of maintenance of NDSs in L22P-expressing cells suggesting that PARP1 is likely required for survival of replication fork stalling and activated in response for stalled forks [46]. It is interesting to compare our results to those seen in previous reports. Replication forks that have been blocked by trapped PARP1 collapse during S-phase resulting in DSBs [47] suggesting that L22P-expressing cells accumulate 5′-dRP groups, which are critical for interaction with PARP1. Our data is in agreement with previous reports that PARP1 forms a covalent bond with 5′-dRP groups and blocks BER [47] or hinders the BER process [48]. Therefore, in the presence of a PARP1 inhibitor, PARP1 can still bind to 5′-dRP group sites in L22P-expressing cells, which eventually causes replication fork collapse.

In conclusion, the results obtained in this study suggest that replication-mediated DSBs are critical to promote genomic instability and cellular transformation in L22P-expressing cells. The present work shows that impairment of the progression of replication forks leads to DSB formation. The dRP lyase function of Pol β is required to minimize the collision of replication forks with 5′-dRP group. In addition, our study shows that POLB polymorphism perturbs the BER pathway and sensitizes cancer cells to PARP1 inhibitors. Our observation may imply that gastric cancer patients carrying defects in POLB function may be stratified for PARP1 inhibitor treatment, resulting in a more effective option.

## MATERIALS AND METHODS

### Cell lines and cultures

The GES-1 gastric epithelial cell line was obtained from the Chinese Academy of Sciences. GES-1 cells were maintained in RPMI supplemented with 10% fetal bovine serum (FBS), 1% glutamate, and 1% penicillin streptomycin.

### Transfection, infection, and expression analysis

Human Pol β and L22P constructs were packaged into retrovirus using the GP2-293 packaging cell line. pRVY-Tet and pVSV-G plasmids were co-transfected into GP2-293 cells using standard calcium phosphate transfection, cells were grown for 72 hours, retrovirus was infect to GES-1 cells, cells were grown to approximately 30% confluence and infected with retrovirus in the presence of 4 μg/ml polybrene. Cells were incubated overnight in fresh media with 4μg/ml polybrene. For selection of pools, cells were split 1:3 the day after infection and cells with the integrated construct were selected with 200μg/ml hygromycin B. Expression of exogenous HA-tagged Pol β was verified by western blot. Cells were passed in parallel in the presence or absence of tetracycline. Approximately 80-90% confluent cells were harvested by scraping with hot SDS Loading Buffer (50 mM Tris pH 6.8, 100 mM DTT, 2% SDS 10% glycerol). Lysates were boiled for 10 minutes and run on a 10% acrylamide SDS-PAGE gel. Proteins were transferred to nitrocellulose membrane using a semi-dry transfer apparatus and probed using monoclonal mouse anti-Pol β antibody (abcam #1831).

### Confocal microscopy of nuclear protein localization, and antibodies used

For γH2AX foci staining, cells were grown on glass cover slips, and fixed with methanol: acetic acid (3:1 ratio), incubated for 15 minutes at −20°C, and permeabilized in PBS containing 0.5% Triton X-100 for 8 minutes at room temperature. Cells were then incubated with 1:200 diluted rabbit polyclonal anti-H2AX antibody (Bethyl Laboratory) for 1 hour at room temperature (RT) and detected with a secondary FITC-conjugated goat anti-rabbit IgG (Jackson Research Laboratory). Antibody dilutions and washes after incubations were performed in PBS containing 0.5% BSA and 0.05% Tween-20. Finally, coverslips were mounted in Vectashield mounting medium with DAPI (H-1500; Vector Laboratories, Burlingame, CA).

### Flow cytometry

Wild type and L22P-expressing GES-1 cells were cultured and treated with 1.5mM MMS for 1 hour. The cell media was removed and the cells were rinsed with 1xPBS then, cells were harvested by trypsinization, washed once with 1xPBS, and pelleted. The pellet was resuspended by adding 70% ice cold ethanol dropwise while vortexing. Cells were fixed overnight at −20°C then incubated with primary phospho-γH2AX antibody (Millipore 05-636) 1:500 overnight at 4°C. Following the incubation, cells were washed twice with1xPBS and incubated with anti-mouse secondary antibody conjugated to FITC 1:500 for 1 hour at room temperature. Cells were washed twice with PBS and resuspended in 500μl PI/RNase staining buffer (BD Pharmingen). Fluorescence was analyzed by flow cytometry using the BD FACSCalibur and analyzed using FlowJo 8.8.6 software.

### Metaphase spreads preparation

For preparation of metaphase chromosome spreads in human L22P-expressing GES-1 cells and WT-Pol β were treated with colcemid (final concentration of 0.1μg/ml) for 6 hours before harvesting. The cells were trypsinized and washed with one time PBS. Mitotic cells, collected and centrifuged at 200×*g* for 5 minutes at room temperature, and the harvested cells were treated with 75mM KCl at 30 minutes at 37°C. After centrifugation, the cells were fixed three times in a freshly prepared mixture of 3:1 methanol: acetic acid. Ten μl of cell suspension were dropped onto slides and allowed to dry, followed by rinsing the slides in phosphate-buffered saline buffer and staining with 5% Giemsa stain for 8 minutes. The slides were rinsed with water and air-dried. Images were acquired with a Ziess microscope.

### Cellular transformation

Anchorage-independent growth was performed as previously described [49]. Briefly, a total of 1×10^4^ GES-1 cells were mixed with media containing 0.7% noble agar (USB) at 42°C. This mixture was poured onto a layer of media containing 1.0% noble agar in a well of a 6-well dish. Cells were grown at 37°C in a 5% CO_2_ humidified incubator and fed twice weekly. The number of colonies present in each of 10 microscope fields per well from a total of 3 wells per experiment were counted after 5 weeks of growth.

### DNA fiber analysis

For DNA replication analysis, sequential labeling of DNA with IdU and CldU were performed based on previously described methods [30]. A sub-confluent, asynchronous population of WT and L22P cells was first labeled for 30 minutes with 25μM IdU, washed with medium three times, and treated with 1.5mM MMS for 1 hour. The cells were then labeled for another 30 minutes with 250μM CldU. After incubation, cells were washed and resuspended at a concentration of 7.5×10^5^ cells/ml. The number of cells lysed per slide ranged between 1500 to 5000 cells using fiber lysis buffer (50mM EDTA, 0.5% SDS, 200mM Tris-HCl, pH = 7.5) for 2 minutes, and the slides were tilted at 20^o^ for gravity flow. The control non-treated cells used were pulsed for 30 minutes with IdU, followed by 1 hour with media only, then pulsed with CIdU label for 30 minutes, and the cells were harvested for the fiber assay. For immunoflourescence staining, the slides were fixed for 10 minutes with methanol: acetic acid (3:1) and air-dried. The slides were treated with 2.5M HCl for 30 minutes, washed with 1xPBS three times, and then blocked with 3%BSA/PBS for 1 hour. CldU was detected by incubating acid-treated fiber spreads with rat anti-BrdU monoclonal antibody (Abcam), and IdU was detected using mouse anti-BrdU monoclonal antibody (1:1000; Becton Dickinson) for 1 hour at room temperature. This was followed by washing three times with 1x PBS and stained with secondary antibody conjugated with sheep anti-mouse Cy3 and goat anti-rat Alexa flour 488 for 1 hour at room temperature. The slides were mounted with Vectashield mounting media and covered with coverslips. Images were acquired with 63x magnification using a Zeiss microscope and processed and analyzed using the ImageJ program. The lengths of red (Cy3) or green (AF 488) labeled patches were measured using the ImageJ software (National Institutes of Health; http://rsbweb.nih.gov/ij/) and arbitrary length values were converted into micrometers using the scale bars created by the microscope. Fluorescence images were captured using a Zeiss LSM 510 inverted confocal microscope using 63×/NA 1.4 oil immersion objective, and data analysis was carried on using the ImageJ software. We applied a conversion factor used is 1 μm = 2.59 kb [50].

### Statistical analysis

All the reported data were evaluated in a pairwise manner: comparing WT versus L22P expressing cells using GraphPad Prism.
